# Sex Differences in Falls: The Mediating Role of Gait Stability Ratio and Body Balance in Vulnerable Older Adults

**DOI:** 10.3390/jcm12020450

**Published:** 2023-01-05

**Authors:** Marcelo de Maio Nascimento, Élvio Rúbio Gouveia, Bruna R. Gouveia, Adilson Marques, Cíntia França, Priscila Marconcin, Duarte L. Freitas, Andreas Ihle

**Affiliations:** 1 Department of Physical Education, Federal University of Vale do São Francisco (UNIVASF), Petrolina 56304-917, Brazil; 2 Department of Physical Education and Sport, University of Madeira, 9020-105 Funchal, Portugal; 3 Laboratory for Robotics and Engineering System (LARSYS), Interactive Technologies Institute, 9020-105 Funchal, Portugal; 4 Center for the Interdisciplinary Study of Gerontology and Vulnerability, University of Geneva, 1205 Geneva, Switzerland; 5 Regional Directorate of Health, Secretary of Health of the Autonomous Region of Madeira, 9004-515 Funchal, Portugal; 6 Saint Joseph of Cluny Higher School of Nursing, 9050-535 Funchal, Portugal; 7 Faculty of Human Kinetics (CIPER), University of Lisbon, 1495-751 Lisbon, Portugal; 8 Faculty of Medicine, University of Lisbon (ISAMB), 1649-020 Lisbon, Portugal; 9 KinesioLab, Research Unit in Human Movement Analysis, Piaget Institute, 2805-059 Almada, Portugal; 10 Centre of Research, Education, Innovation and Intervention in Sport (CIFI2D), Faculty of Sport, University of Porto, 4200-450 Porto, Portugal; 11 Department of Psychology, University of Geneva, 1205 Geneva, Switzerland; 12 Swiss National Centre of Competence in Research LIVES–Overcoming Vulnerability: Life Course Perspectives, 1015 Lausanne, Switzerland

**Keywords:** sex differences, aging, gait pattern, vulnerability, postural control, falls

## Abstract

This study, conducted on a large sample of older adults at elevated fall risk (1), aimed to verify statistical differences in gait stability ratio (GSR) and body balance (BB) according to sex, (2) to examine and compare GSR and BB performance between older adult fallers and non-fallers, (3) to determine an association between GSR and BB according to the history of falls, and (4) to explore whether GSR and BB mediate the association between sex and falls. We included 619 individuals (69.8 ± 5.6 years) living in the Autonomous Region of Madeira, Portugal. The frequency of falls was obtained by self-report. BB was determined by the Fullerton Advanced Balance scale, while GSR was established by dividing cadence by gait speed and data collected during the 50-foot walk test. Males indicated a lower prevalence of falls in the last 12 months (23.6%), while females had a higher score (48.7%), as well as a lower balance performance (*p* < 0.001) and higher GSR scores (*p* < 0.001). Lower BB control (*p* < 0.001), as well as higher GSR, were more expressive for fallers (*p* < 0.001). We found a large, negative and significant correlation between GSR and BB for historical falls (*r* = −0.560; *p* < 0.001), and between male and female cohorts (*r* = −0.507; *p* < 0.001). The total effect of sex on falls mediated by GSR and BB was 16.4%. Consequently, GSR and BB mediated this association by approximately 74.0% and 22.5%, respectively.

## 1. Introduction

Among the vulnerable older adult population, falls are the most common cause of accidents [1], and their prevalence is directly proportional to age [2]. Falls events are often followed by injuries [3] and hospitalization days [4], and notwithstanding, may limit the performance of the activities of daily living [5]. Depending on the severity of the event, its consequences can lead to death [6,7]. Falls lead the causes of morbidity and transmit a great burden to the individual, family, and society [8]. For these reasons, falls are considered a serious public health problem [9]. It is estimated that at least a third of people aged 65 and over fall once over a year, and half of those more than once [10]. In turn, these episodes generate huge costs for public and private health services [11,12]. The literature highlights that falls have multifactorial origins [13,14], which can be categorized according to extrinsic and intrinsic risk factors. Examples of extrinsic factors are environmental issues such as lighting, flooring, placement of objects in space, or socioeconomic components (i.e., income, housing status, and years of schooling). Intrinsic factors are those generated by behavioral issues (i.e., attitudes, medications) or biological issues such as age, comorbidities, physical abilities, and somatosensory, vestibular, and visual systems.

In the case of the older adult population, falls have a particularity, which is their incidence according to sex [15]. Studies suggested that females were more likely to fall than males because they were more afraid of falling, had less confidence in their balance, and were more exposed to domestic accidents because they were more active than males [15,16]. A population-based study in the US found that fall-related injury rates among females were 40–60% higher than among males [17]. According to the authors, the hospitalization rate for injuries was 81% higher for females. In another population-based study conducted in Canada [18], the prevalence of falls found for females and males was 22.4% and 17.3%, respectively. Comparatively, the rate of medical care needed by females was 7.2%, while for males it was 4.2%. Some investigations have reported a higher prevalence of falls due to a greater predisposition of males to diseases, which are accompanied by a higher consumption of medications, which in turn can alter gait and balance performance [18,19]. In addition, evidence has been presented that there is no association between falls and sex [20]. For these reasons, the literature has shown that the factors associated with an increased risk of falls according to sex are not yet fully understood [17]. Notably, the facts point to the need to deepen the understanding of the factors associated with falls according to the sex of the fallers. From this, it is possible to extend this issue to two important points: the creation of methods to identify the risks of falls and the improvement of fall prevention programs specific to each sex [21].

Among the intrinsic factors associated with an increased risk of falling in older adults are gait speed (GS) and body balance (BB), both of which are susceptible to the physiological changes of aging. This means that older adults are less able to plan body stabilization strategies, which depend on physical functions [22,23]. It is known that from the age of 60, both the GS [24] and the control of BB continually change [25]. GS, for example, decreases by about 1% per year from 60 years old [26]. Moreover, after age 70, gait disturbances can increase by up to 35% [27]. In turn, physiological ageing causes changes in the functioning of the visual, vestibular, and somatosensory systems, which are responsible for regulating static and dynamic BB [28,29]. For this reason, older adults are constantly forced to seek strategies to stabilize posture and voluntary body movement planning, including gait stability [30].

Generally, most older adult fall events occur during body movement [31]. Postural deficits in older adults have been associated with reduced skeletal mass [32], and decreased lower limb strength [33]. One strategy used by older adults to adapt their movement pattern consists of gait deceleration [34], accompanied by a decrease in stride length [35]. With this, older adults achieve a lower forward progression of the body [36], reducing the risk of falling with each step. Cromwell et al. [37] suggested a measure capable of reflecting variations in stride length, which considers changes in GS. This measure is the gait stability ratio (GSR), obtained by the ratio between cadence (steps/s) and speed (m/s). An advantage of the GSR measure is the indication of gait instability based on the increase in the GSR value. Therefore, the more steps per unit of distance an older adult performs, the greater the contact time of the lower limbs with the ground. This strategy reduces the dynamic components of gait, making gait and posture more stable [36].

In a previous yet contemporary publication [37], carried out by the research group of the present manuscript in a large population of older adults, associations between GS, cadence (CAD), GSR, and BB with falls were revealed. The findings showed that participants classified as GS and BB in the lowest tertile indicated an increased chance (OR) of falling by approximately 149.3% and 48.8%, respectively. In addition, compared to the highest tertile, older adults with GSR values categorized in the lowest and middle tertiles indicated an increased chance of falling by up to 57.4% and 56.4%, respectively. Therefore, the lower the gait variability, the lower the chance of falling. In another mediation study developed by the same research group mentioned [38], the authors verified whether lower limb muscle strength (LEMS) and BB performance mediated the relationship between physical activity level (PA) and health-related quality of life (HRQoL). The results indicated that LEMS and BB mediated the association between PA and HRQoL by approximately 39.6% and 47%, respectively. All these findings generated new research questions, such as whether there would be differences between the sexes in variables such as BB and GSR in the context of falls. To the best of our knowledge, no study has investigated in depth the relationship between these two variables.

Thus, to address these important gaps in the previous literature, the present study, conducted with a large sample of older adults, aim (1) to verify the statistical difference in GSR and BB according to sex, (2) to examine and compare GSR and BB performance between older adult fallers and non-fallers, (3) to determine the association between GSR and BB according to the history of falls, and (4) to explore whether GSR and BB mediate the association between sex and falls. It is worth mentioning that in the present study, the term sex was assumed to be a biological attribute of the participants [39]. However, it should be considered that differences between the sexes involve physical and behavioral differences, referring to a series of social and cultural contexts, which in turn determine the gender (i.e., man and woman) of an individual [40].

## 2. Methods

### 2.1. Design and Participants

A cross-sectional analytical observational study was carried out with 619 individuals (69.8 ± 5.6 years). Of these, 305 were male, and 314 were female. All participants resided in the Autonomous Region of Madeira, Funchal, Portugal. The study was publicized in the local press, as well as through pamphlets in cultural and sports associations, squares, churches, and directly in homes. Inclusion criteria were as follows: (1) residing in the community of Madeira (Funchal), (2) age between 60 and 79 years, and (3) ability to walk independently without assistance. The exclusion criteria adopted were: inability to follow the assessment protocols and medical contraindications to perform submaximal exercises according to the guidelines of the American College of Sports Medicine [41]. The Scientific Committee of the Department of Physical Education and Sports of the University of Madeira scientifically and ethically approved our study, as did the Regional Secretariat of Social Affairs Committees. Additionally, we received support from the FCT (SFRH/BD29300/2006). The procedures took place following the procedures of the declaration of Helsinki. Before participating in the assessments, all older adults were informed about the activities and signed a consent form. All data were collected in 2009 by a team of researchers specially trained at the University of Madeira (Laboratory of Human Physical Growth and Motor Development).

### 2.2. Data Collection

#### 2.2.1. Demographic and Health Characteristics

Sociodemographic and health information, such as sex, age, comorbidities, and the number of medications consumed daily were collected through face-to-face interviews. The procedures were performed by previously instructed field team members.

#### 2.2.2. Falls

Information on the participants’ history of falls was collected through face-to-face interviews, with the following questions: (1) In the last 12 months, have you ever fallen? Answers: yes/no; (2) If yes, the interview continued with the following question: Do you remember how many times you fell in the last 12 months? (3) Subsequently, the fear of falling was investigated. The question was: How do you rate your fear of falling on a scale from zero to four? The results were interpreted as follows: zero absence for fear of falling, and four as maximum fear of falling.

#### 2.2.3. Anthropometry

Height and body mass were measured using an anthropometric scale and a Welmy^®^ (London, UK) stadiometer, coupled with 0.1 cm and 0.1 kg [42]. Body mass index (BMI) was determined by the equation (weight [kilograms])/(height [m]^2^). All measurements were performed by previously trained examiners.

#### 2.2.4. Gait Parameters

Gait parameters of interest were GS, CAD, and GSR. GS (m/s) was assessed using the 50-foot walk test [43]. This gait test showed high test–retest reliability for households (0.94), as well as an acceptable coefficient of validity for males (0.79) and females (0.80). The best performance among the three attempts was assumed. Participants were asked to walk a distance of 15 m at their usual speed. The GS performance was performed by dividing the distance covered during the 50-foot walk by the time used until the end of the test. The CAD (steps/s) was obtained by dividing the number of steps performed over 50 foot by the time used to complete the task. The number of steps taken over the 50-foot distance was counted by visual observation. The calculation of the GSR (steps/m) was performed by dividing the CAD performance by the GS performance [36]. Previous studies have shown GS, CAD, and GSR to be auxiliary and effective measures, capable of identifying the mechanisms used by older adults to adjust gait and maintain stable dynamic balance [44,45].

#### 2.2.5. Balance

BB was assessed using the Fullerton Advanced Balance (FAB) scale [46]. Based on ten tasks, the FAB evaluates older adult individuals’ static and dynamic balance performance. Its evaluation system uses on an ordinal scale from 0 (zero) to 4 (four) points, with a maximum performance of 40 points. Its predictive validity concerning the risk of falling has been previously presented [47]. FAB tasks are as follows: (1) stand with feet together and eyes closed; (2) with arm outstretched, reach out to pick up an object (pencil) at shoulder height; (3) perform a 360° turn to the left and then to the right; (4) perform movements up and down (15 cm bench); (5) tandem walk; (6) stand on one leg; (7) stand on one side on foam with eyes closed; (8) jumping from a distance; (9) walk and simultaneously move the head, directing vision to the right and left side; and (10) recover an unexpected loss of balance. A detailed description of the FAB administration protocol and the necessary equipment and instructional video can be accessed [46]. There was a score of 25/40 yields the highest sensitivity (74.6%) and specificity (52.6%) in predicting fall risk [48]. The FAB showed excellent test–retest reliability (ICC= 0.98), excellent test re-test reliability (*r* = 0.96) for a total score, as well as acceptable internal consistency (ᾳ > 0.75) for all 10 items [46].

#### 2.2.6. Covariates

In the present study, the following variables were assumed as possible confounding factors and, therefore, controlled in the serial analysis of mediation: age, number of medications consumed daily, musculoarticular problems, and BMI. Although these variables were not of interest to the investigation, they played an important role in physiological aging [49,50], affecting the analysis mediators (GSR, BB), and in turn, our response variable (falls).

#### 2.2.7. Statistics

Data distribution was examined using the Kolmogorov–Smirnov test. After verifying normal distribution, continuous variables (i.e., age, medications, weight, height, number of falls in 12 months, BMI, GS, CAD, GRS, BB) were presented as mean and standard deviation (SD) values. Categorical variables (i.e., sex, and comorbidities) were presented through frequencies and percentages. The primary and secondary objectives of the study were verified via the unpaired Student’s *t*-test for independent samples. The procedures examined significant differences between continuous variables (GSR and BB) and dichotomous variables (sex and history of falls). For the third objective, Pearson’s correlation analysis was used, which tested the strength and direction of the relationship between GSR and BB, according to the participants’ history of falls. The adopted correlation coefficients (*r*) were the following: 0.1 = small, 0.3 = medium, and ≥0.5 = large [51]. Finally, we proceed with an analysis of mediation (the fourth objective of the investigation). In this analysis, the dependent variable falls (*y*) was composed of the total number of falls (scalar variable) in the last 12 months. This variable was extracted from the self-report obtained in a face-to-face interview.

**Figure 1:**
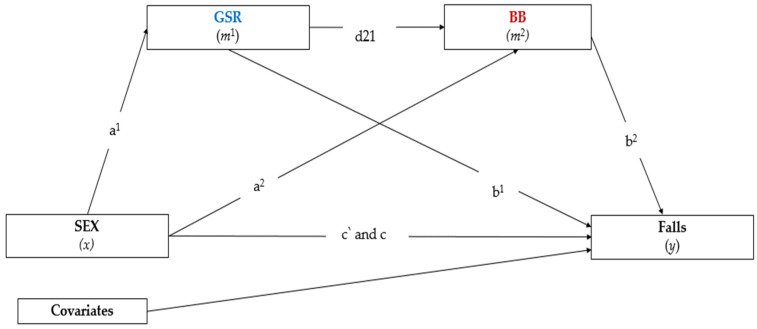
Path (a^1^) = association between independent variable sex (*x*) with GSR mediator (*m*^1^), path (a^2^) = association between independent variable sex (*x*) with BB mediator (*m*^2^). Path (b^1^) = association between mediator GSR (*m*^1^) with dependent variable Falls (*y*), path (b^2^) = association between mediator BB (*m*^2^) with dependent variable Falls (*y*), and path (d21) = association between both mediators GSR and BB (*m*^1^ and *m*^2^). Path (c’) = association between independent variable Sex (*x*) with dependent variable Falls (*y*), and Path (c) = total indirect effect through paths a^1^, b^1^, a^2^, b^2^, and d21.

Before carrying out the procedures of the mediation analysis, we verified the assumptions of the multiple regression analysis: the linear relationship between independent and dependent variables, mean of residuals equal to zero, normality of residuals, non-multicollinearity, non-autocorrelation of residuals, homoscedasticity of residuals, or equal variation [52]. Figure 1 presents the assumed path model, composed of five indirect effects (a^1^, b^1^, a^2^, b^2^, d21), the direct effect (*x*–*y* paths), and the total indirect effect through pathways a^1^, b^1^, a^2^, b^2^, d21. Our model included two parallel mediators (*m*^1^ and *m*^2^). In general, our analysis was composed of two models: the first was responsible for the paths between *x*-*m*^1^ and *x*-*m*^2^, while the second represented the paths between *m*^1^-*y* and *m*^2^-*y*. This type of analysis both broadens and qualifies the understanding of how an independent variable (sex) can directly influence a dependent variable (falls), mediated in parallel series by two mediators (GSR and BB). In such a way, a complete mediation will be observed if, with the inclusion of objectively measured GSR and BB (mediator variable), the association between the independent variable (sex) and the dependent variable (falls) does not remain significant. If this occurs, the confidence interval obtained will include the value zero [53]. On the other hand, a partial mediation was considered in the event that the observed relationship between the independent variable (sex) and the dependent variable (falls) becomes weaker after the inclusion of the mediating variables (*m*^1^ and *m*^2^).

We used PROCESS v4.0, which is a tool of the SPSS program, to estimate regression coefficients. The mediation analysis was processed by Model 6 [53]. All coefficients described in the equation (Figure 1) were estimated using least-squares regression analyses. Thus, the mediation hypotheses were calculated by the confidence interval (95% CI), with the correction of bias and acceleration (BCa) determined by the bootstrap method. The number of bootstrap samples was set at 5000. Specific indirect effects were accepted as significant when the confidence interval did not include zero [54]. The effects of mediation proportions were calculated by subtracting 1 minus the result of dividing the direct effect and the total effect [53]. The results illustrated in Figure 1 are equivalent to the standardized parameters (*β*). The significance level adopted for all analyses was *p* < 0.050.

## 3. Results

### 3.1. Main Characteristics of Participants

The study consisted of 619 older adult individuals of both sexes (see Table 1 for an overview). Of these, 314 were female. The mean age of the participants was 69.8 ± 5.6 years. Regarding the history of falls in the last 12 months, 225 (36.3%) were fallers. The prevalence of falls among females was 48.7% (*n* = 153), while among males it was 23.6% (*n* = 72) (*p* < 0.001). The daily consumption of different types of medication was 3.85 ± 1.97, a result which indicated the statistical difference between males and females (*p* = 0.048). Among the self-reported comorbidities, there was a general prevalence and statistically different results (*p* ≤ 0.050) between genders for the following variables: visual impairment (61.0%) and hypertension (49.7%), followed by hearing (30.8%), diabetes (23.9%), and musculoskeletal (5.7%) problems. Regarding the objectively measured motor variables, comparatively, males performed better in all tests. For GS males, variables indicated a performance of 1.29 ± 0.24, while females reached a mean score of 1.22 ± 0.24 (*p* < 0.001). Females had a higher mean score of 1.94 ± 0.22 CAD, while males had a mean score of 1.89 ± 0.22 (*p* = 0.004).

### 3.2. GSR and BB Performance According to Sex and History of Falls

**Figure 2:**
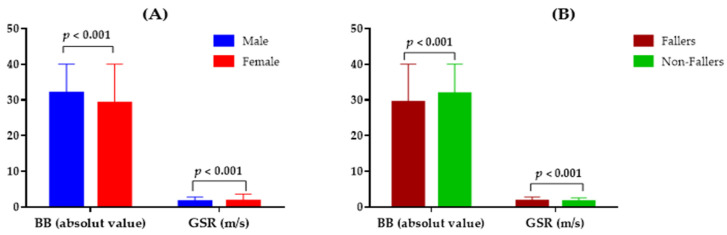
Comparative graph of GSR values and BB performance, according to sex and history of falls. m/s = meters per second. (**A**) Results by sex; (**B**) Results according to historical declines in the last 12 months.

Figure 2A shows the performance of BB and GSR according to sex. Comparatively, males had a superior BB performance of 31.94 ± 6.11 (*p* < 0.001), while females had a score of 29.13 ± 6.38 (*p* < 0.001). On the other hand, concerning the GSR, males showed lower values 1.51 ± 0.22 (*p* < 0.001), and females indicated a superior score of 1.57 ± 0.22 (*p* < 0.001). Figure 2B comparatively presents the BB performance and the participants’ GSR score according to the history of falls. The fallers indicated a lower BB performance of 29.41 ± 6.23 (*p* < 0.001), while the non-fallers showed a higher average performance of 31.80 ± 6.38 (*p* < 0.001). Regarding the GSR, fallers indicated a higher value of 1.63 ± 0.22 (*p* < 0.001), and non-fallers had a lower mean value of 1.52 ± 0.22 (*p* < 0.001).

### 3.3. Results of the Correlation Coefficients between GSR and BB According to the History of Falls and Sex

**Figure 3:**
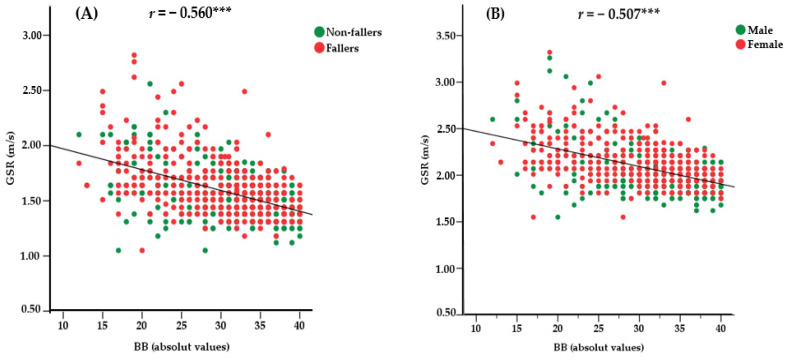
Scatterplot of correlation between GSR and BB performance. m/s = meters per second. (**A**) Results according to the history of falls in the last 12 months; (**B**) Results by sex; *** *p* < 0.001.

Figure 3 shows the scatterplot of the correlation analysis between GSR and BB. Figure 3A presents the result of the analysis between GSR and BB for fallers and non-fallers: the verified correlation was large, negative and significant (*r* = −0.560; *p* < 0.001). When analyzing according to sex (Figure 3B), we also obtained a large, negative and significant correlation for GSR and BB (*r* = − 0.507; *p* < 0.001).

### 3.4. Mediation Analysis

**Figure 4:**
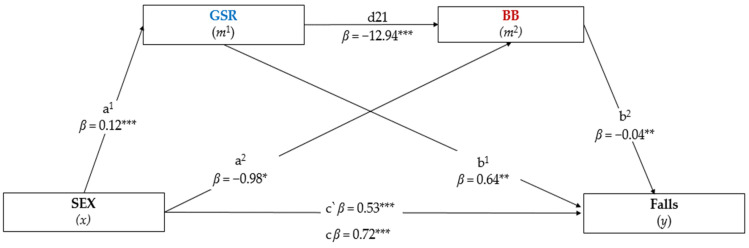
Parallel mediation analysis of the effects of sex (independent variable) on falls (dependent variable) through GSR (gait stability ratio) and BB (body balance). The analysis was based on 5000 bootstrap samples. The indirect effect was statistically significant at the 95% confidence interval (CI) when the CI did not include 0 (zero). Betas (*β*) were reported as the product of simultaneous regression with bootstrap replacement: (1) Path a^1^ and a^2^ = association between sex with GSR and BB, respectively, (2) Path d21 = association between the two mediation variables (*m*^1^ and *m*^2^), (3) Path b^1^ and b^2^ = association between GSR and BB with falls, (4 Path c, = direct effect (*x*–*y*): associations *m*^1^ blue and *m*^2^ dark red = indirect effect (*x*–*y*) by GSR and BB, respectively. * *p* < 0.050, ** *p* < 0.010, *** *p* < 0.001, c = total effect; c’ = direct effect; a = path Model 1; b = Model 2 path.

The general model obtained was significant: F(2613) = 49.626, *p* < 0.001, R^2^ = 0.37, predicting GRS and BB to be mediators of the relationship between sex and falls (Figure 4). Model 1 was controlled for confounders (i.e., age, number of medication, musculoarticular problems, and BMI), indicating that sex (independent variable) had a positive and significant association with the GSR mediator (*β* = 0.12, *t*(613) = 7.114, *p* < 0.001), but a negative and significant association with the BB mediator (*β* = −0.98, *t* (613) = −2.000, *p* = 0.032). The estimated association between both mediators (*m*^1^–*m*^2^) was significant and negative (*β* = −12.94, *t*(612) = −12.078, *p* < 0.001). Model 2 indicated significant and positive associations between the GSR mediator (*β* = 0.64, *t*(611) = 2.062, *p* = 0.032) with falls (dependent variable), on the other hand, the mediation effect between BB and falls was negative and significant (*β* = −0.04, *t*(611) = −3.717, *p* < 0.002). When the mediating variables (*m*^1^ and *m*^2^) were included, the path estimated by the model remained significant. Thus, the direct effect, estimated by the model (*x–y*), indicated a positive and significant relationship between sex and falls (*β* = 0.53, *t*(613) = 4.175, *p* < 0.001). The total effect of the model (*x*–*y*) observed was also positive and significant (*β* = 0.72, *t*(613) = 5.719, *p* < 0.001). In our serial mediation path model, three effects were tested: (1) the indirect path through GSR was positive and significant (*β* = 0.08, *SE* = 0.042, 95% CI BCa = 0.0013–0.1694), (2) the specific indirect effect through BB was also positive and significant (*β* = 0.039, *SE* = 0.025, 95% CI BCa = 0.0003–0.0577), and (3) we concluded that GSR and BB partially mediated the general indirect pathway from sex to falls via a positive and significant association (*β* = 0.0641, *SE* = 0.029, 95% CI BCa = 0.0159–0.1290). These findings indicated that GSR and BB were independent mediators of the effect that sex has on fall events in community-dwelling older adults. Finally, the proportion of the total effect of sex on falls mediated by GSR and BB was 16.4%. GSR explained approximately 74.0% of the variance of the association between sex and falls, while the proportion exercised by BB in the relationship was 22.5%.

## 4. Discussion

Our study had four objectives. First, we verified whether the GSR value and the BB performance would indicate statistical differences in the comparison between the sex of the participants. We found that females reported lower balance performance than male participants and had higher GSR scores. The findings also corroborated the previous literature [55,56], proving a higher prevalence of falls for females (48.7%) compared to males (23.6%). Second, we examined whether the GSR and BB measures would indicate statistical differences in comparing fallers and non-fallers. A lower ability of postural control, as well as a high value of GSR, was more conclusive for the group of fallers. This finding was in line with the previous literature [36]. Our third objective was to determine the association between GSR and BB according to the history of falls. Confirming that high GSR values are associated with poor postural control [45,57], a large, negative and significant association was found. This result suggested the GSR is a predictor of falls in the older adult population, as was found in previous studies [58,59].

The fourth objective was to explore whether GSR and BB mediated the association between sex and falls. When BB and GSR were placed concomitantly in the equation as mediators, and the results were controlled for covariates (i.e., age, number of medications, musculoskeletal problems, and BMI), the effects of the direct and total trajectory between *x*–*y* remained significant. Thus, in proportional terms, BB and GSR partially mediated the association between sex and falls, at approximately 22.5% and 74.0%, respectively. These findings may contribute to understanding the negative role that a high GSR value plays in postural control, in addition to suggesting how much gait variability is associated with BB control. Finally, the model’s total variance explained by sex in the relationship with falls was 16.4%. This result is representative, mainly because falls have multifactorial causes [24]. They arise from intrinsic and extrinsic factors [2]. As far as we know, the present study is the first one to analyze this mediation mechanism.

Our results align with previous studies that attested the temporal and spatial variability of gait as associated factors responsible for changes in mobility and increased risk of falling in older adults [60,61]. Moreover, we showed that deficits in gait variability and BB were more conclusive for females. The literature highlights that there have been gaps in the relationship between sex and falls, e.g., why falls are prevalent among females [62,63]. Among the recurring explanations for the frequency of falls and injuries among females are the biological differences and behavioral stratification between sexes [15]. In a cohort study that included 276 males (78.3 ± 5.2 years) and 467 females (78.1 ± 5.0 years) in order to investigate external and internal circumstances, as well as the consequences of falls [64], the authors concluded that males had a greater number of injuries occurring outdoors during recreational and vigorous activities. Conversely, females presented a higher risk of falling when performing less vigorous activities at home.

These findings highlight the need to consider the particularities of the sexes in the context of older adult population falls. Furthermore, they suggest the importance of developing sex-specific strategies for preventing falls [1], and the need to make the vulnerable older adult population aware of the risk factors associated with falling in each sex [65]. Among the possible measures, there is the voluntary participation of the older adult in lectures, the creation of booklets and educational videos [66], as well as adjusting the training programs (i.e., exercises) to the challenging situations of the daily life of each sex [17]. Moreover, it should be considered that males and females perceive falls differently [21], which can substantially influence their involvement in fall prevention programs.

An important point to highlight is that GSR values can provide information about the cognitive status of healthy individuals. Among older adults, the regulation of gait variability is automated by a minimal cognitive input system [67]. This requires the involvement of higher executive control processes [68]. Thus, increased stride length variability may be associated with subclinical cerebrovascular abnormalities [69]. Therefore, our findings may help to develop therapeutic implications because, even in healthy older adults, mild neurodegenerative processes can generate changes in gait regulation [70].

The population of the present study was composed of cognitively healthy individuals. However, it is known that gait changes may indicate some executive dysfunction [71,72]. On the other hand, gait parameters such as speed, cadence and step width can benefit from properly structured interventions [73,74]. Moreover, depending on the type of physical training [75,76], it is possible to simultaneously improve gait and balance and promote neural plasticity [77,78]. Another point to be addressed concerning sex and falls is that studies have attributed a greater fear of falling to females (FoF) [16,79]. A possible explanation is an awareness and/or concern that females have. In this scenarios, females know that they are more likely to fall in relation to males [80]. However, in the present study comparing the sexes, FoF did not indicate a significant difference for sex.

Our study has some limitations. First, its cross-sectional design does not allow us to draw conclusions regarding the direction of the cause-and-effect relationship between sex and falls. However, the statistical procedure used (mediation analysis) is considered a robust approach, which allows the inference of potential causalities [81,82]. Second, the participants in this study were recruited from different locations, and so they may have had different levels of physical activity. Therefore, cases of a sedentary lifestyle may have generated inter-individual differences in BB’s performance and GS and CAD results, consequently reflecting the calculated GSR value. Third, FoF was assessed with a single and non-specific questionnaire. Thus, it is suggested that future studies examine the perception of FoF through validated and widely used scales. A strong point of this investigation was the representative sample, formed by many older adults residing in a defined geographic area (Madeira Island). In health, sectoral information is essential for controlling lifestyle habits and diseases, contributing to creating effective policies [83]. In terms of future investigations, it is suggested to examine the relationship between sex and falls, mediated by other variables, such as motor skills (i.e., lower limb strength, coordination, agility), sociodemographic factors (i.e., age groups), cognitive performance, lifestyle, comorbidities, or FoF.

## 5. Conclusions

From two objective measures of quick access and low cost (GS and CAD), it was possible to evaluate GSR and, consequently, to detect important information about falls in the population studied. Our findings suggested that GSR and BB partially mediated the relationship between sex and falls by up to 74.0% and 22.5%, respectively. Considering that falls are multifactorial events, these results are significant and can help understand and treat a severe public health problem. We found that the increase in gait variability (GSR) was strongly associated with a greater degree of imbalance, represented by the faller group, and also with being female. Finally, the information provided by this study can expand the understanding of the use of GSR, which proved to be a useful tool for detecting strategies used by vulnerable older adults at elevated fall risk to adapt their gait to their dynamic balance deficit, preventing falls.

## Figures and Tables

**Table 1:** Main characteristics of the sample.

Variable	Full Sample (*n* = 619)	Female (*n* = 314)	Male (*n* = 305)	*p* Value
Age (years)	69.50 ± 5.62	69.49 ± 5.57	69.51 ± 5.70	0.960
Age group *n* (%)				0.842
60–69 years	294 (47.5)	145 (46.2)	149 (48.8)	
70–79 years	303 (48.9)	158 (50.3)	145 (47.5)	
80–89 years	22 (3.5)	11 (3.5)	11 (3.6)	
Falls (%)				<0.001
Yes	225 (36.3)	153 (48.7)	72 (23.6)	
No	394 (63.7)	152 (48.4)	242 (79.3)	
Number of falls (*n*)				<0.001
1–2	164 (72.9)	104 (33.1)	60 (19.7)	
3–4	38 (16.9)	31 (9.8)	7 (2.2)	
5–6	15 (6.7)	13 (4.1)	2 (0.6)	
7–10	8 (3.6)	5 (1.6)	3 (0.9)	
Fear of falling (*n*)	2.21 ± 0.50	2.44 ± 0.84	2.48 ± 0.72	0.202
Medication (*n*)	3.85 ± 1.97	4.10 ± 1.32	4.71 ± 1.01	0.048
Height (cm)	159.05 ± 8.69	152.94 ± 5.72	165.64 ± 5.73	<0.001
Weight (kg)	74.77 ± 13.06	70.92 ± 12.14	79.22 ± 12.45	<0.001
BMI (kg/m^2^)	29.51 ± 4.34	30.28 ± 4.73	28.80 ± 3.86	<0.001
Hypertension	308 (49.7)	162 (51.6)	146 (47.8)	0.041
Diabetes	148 (23.9)	56 (17.8)	92 (30.1)	0.028
Visual impairment	378 (61.0)	284 (90.4)	202 (66.2)	0.032
Hearing problems	191 (30.8)	89 (28.3)	107 (35.0)	0.029
Musculoarticular problems	46 (5.7)	24 (7.6)	22 (7.2)	0.049
GS (sec.)	1.26 ± 0.25	1.22 ± 0.24	1.29 ± 0.24	<0.001
CAD (sec.)	1.91 ± 0.22	1.94 ± 0.22	1.89 ± 0.22	0.004

BMI: body mass index; CAD: cadence; GS: gait speed; sec.: seconds; *p* < 0.05.

## Data Availability

The data presented in this study are available upon request from the corresponding author.
